# Supplementary material for: Human Sex Matters: Y-Linked Lysine Demethylase 5D Drives Accelerated Male Craniofacial Osteogenic Differentiation
Source: Cells. 2022 Feb 26;11(5):823. doi: 10.3390/cells11050823 (PMC8909072; doi:10.3390/cells11050823)
Supplement: Supplementary file 1 [file cells-11-00823-s001.zip › cells-1508708-supplementary.pdf]

cells

MDPI

Article

Human Sex Matters: Y-Linked Lysine Demethylase 5D Drives Accelerated Male Osteogenic Differentiation

Madlen Merten[1,#], Johannes F.W. Greiner[2,3,#], Tarek Niemann[1,3], Meike Grosse Venhaus[1], Daniel Kronenberg[4], Richard Stange[4], Dirk Wähnert[3,5], Christian Kaltschmidt[2,3], Thomas Vordemvenne[3,5,§] and Barbara Kaltschmidt[1,2,3,§,*]

Supplemental Material

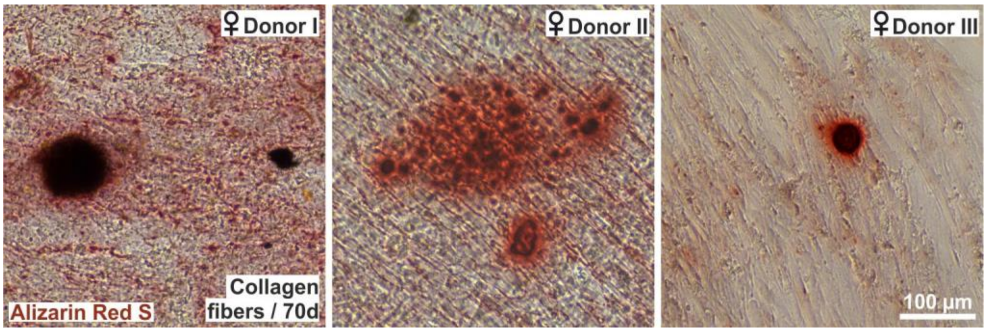

Figure S1. NCSCs from female donors showed Alizarin Red S-stained calcium deposits after culture on collagen fibers for 70 days.

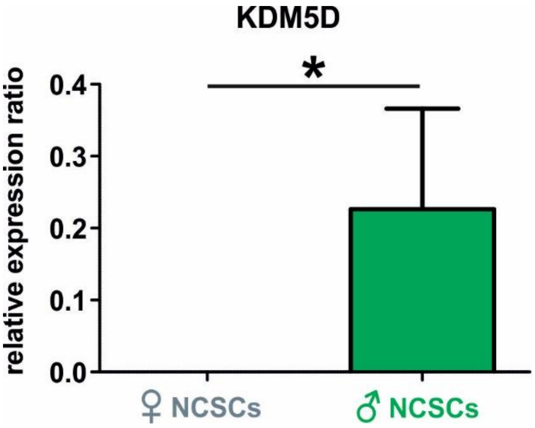

Figure S2. Validation of RNA-Seq analysis via qPCR showing a significantly increased expression of KDM5D in male NCSCs compared to female NCSCs after 30 days of differentiation. Biological triplicate (NCSCs from three male and three female donors), Mann Whitney test, $^{*}P < 0.05$ was considered significant.
